# Discovery and insights from DSX mission’s high-power VLF wave transmission experiments in the radiation belts

**DOI:** 10.1038/s41598-022-18542-9

**Published:** 2022-08-22

**Authors:** P. Song, J. Tu, I. A. Galkin, J. P. McCollough, G. P. Ginet, W. R. Johnston, Y.-J. Su, M. J. Starks, B. W. Reinisch, U. S. Inan, D. S. Lauben, I. R. Linscott, W. M. Farrell, S. Allgeier, R. Lambour, J. Schoenberg, W. Gillespie, S. Stelmash, K. Roche, A. J. Sinclair, J. C. Sanchez

**Affiliations:** 1 Space Science Laboratory, University of Massachusetts Lowell, Lowell, MA USA; 2 Department of Physics and Applied Physics, University of Massachusetts Lowell, Lowell, MA USA; 3 Space Vehicles Directorate, Air Force Research Laboratory, Kirtland AFB, Albuquerque, NM USA; 4 Department of Energy, Albuquerque, NM USA; 5 MIT Lincoln Laboratory, Lexington, MA USA; 6 Department of Electrical Engineering, Stanford University, Palo Alto, CA USA; 7 Department of Electrical Engineering, Koç University, Istanbul, Turkey; 8 Goddard Space Flight Center, NASA, Greenbelt, MD USA

**Keywords:** Astronomy and planetary science, Space physics, Magnetospheric physics, Astrophysical plasmas

## Abstract

Space weather phenomena can threaten space technologies. A hazard among these is the population of relativistic electrons in the Van Allen radiation belts. To reduce the threat, artificial processes can be introduced by transmitting very-low-frequency (VLF) waves into the belts. The resulting wave-particle interactions may deplete these harmful electrons. However, when transmitting VLF waves in space plasma, the antenna, plasma, and waves interact in a manner that is not well-understood. We conducted a series of VLF transmission experiments in the radiation belts and measured the power and radiation impedance under various frequencies and conditions. The results demonstrate the critical role played by the plasma-antenna-wave interaction around high-voltage space antennae and open the possibility to transmit high power in space. The physical insight obtained in this study can provide guidance to future high-power space-borne VLF transmitter developments, laboratory whistler-mode wave injection experiments, and the interpretation of various astrophysical and optical phenomena.

## Introduction

Space weather phenomena^1^ threaten the space assets that bring us services via space technologies, such as the Global Positioning System, communication systems with satellite relays, and most global TV broadcast networks, which have provided unprecedented convenience to everyday life and opportunities to businesses. A hazard among the space weather phenomena is the population of relativistic electrons in the region called the Van Allen radiation belts^2^. These electrons can be trapped for years once produced by either natural^3^ or artificial processes^4^ and can damage the electronics and degrade the solar panels on satellites. Intense investigations on this issue have continued, for example, via the NASA satellites Van Allen Belt Probes A and B in 2012–2019^5–13^. To remedy the threat and reduce the resulting damage, artificial processes can be introduced to shorten the lifetime of these particles^14^ with mechanisms such as pitch-angle diffusion through wave-particle interactions^15–17^, which may deplete the harmful electrons and make them precipitate into the neutral atmosphere. The most effective waves for this process are in the frequency range called the whistler-mode in plasma physics^2^ and the very-low-frequency (VLF) waves in electrical engineering. High-power terrestrial VLF transmitters are large and expensive but well-understood. However, most of the wave power will not be able to propagate through the ionosphere reaching the radiation belts, instead, it stays and eventually is absorbed in the ionosphere.

Ideally, it is most efficient to directly transmit the VLF waves in the radiation belts. However, this is an extremely challenging task, mostly because the processes associated with the wave-antenna-plasma interaction are not well-understood. Several models have been proposed, including the conventional vacuum model^18^, theoretical approaches based on the reaction method^19–21^, and models developed during recent investigations^22–24^. The differences in predictions of the radiation impedance among these models and numerical simulations can be as large as five orders of magnitude! Without knowing the range of the radiation impedance, an efficient VLF transmission system cannot be designed. The only way to resolve this problem is via direct experiment by launching a satellite into the radiation belts with the capability of transmitting VLF waves in an extremely large range of possible radiation impedance.

The U.S. Air Force Research Laboratory’s Demonstration and Science Experiments (DSX) satellite was designed to study the processes and was launched on June 25, 2019, into an orbit that passes through the radiation belts^25^. On the satellite is a transmitter with an 82-m tip-to-tip dipole antenna. In order to transmit high power, the novel transmitter, named Transmitter-Narrowband receiver-Tuner (TNT), carries a tuner that is able to automatically tune to a desired resonance frequency according to the plasma conditions which change rapidly as the satellite moves in space. After the successful deployment of the antenna in July 2019, TNT conducted a large number of successful transmission experiments. The mission concluded on May 31, 2021.

In this initial study, we report the most comprehensive measurements yet acquired of radiation impedance from high-power VLF wave transmission experiments in the radiation belts. The discoveries from the experiments can be used to identify key physical processes and develop, test, and validate theoretical models of whistler-mode transmission. They can also help shed light on many processes in space, astrophysics, and optics. In addition, the physical understanding obtained in this study may provide a guide to laboratory whistler-mode wave injection experiments^26^, e.g., in controlled fusion research^27^.

## Results

**Figure 1:**
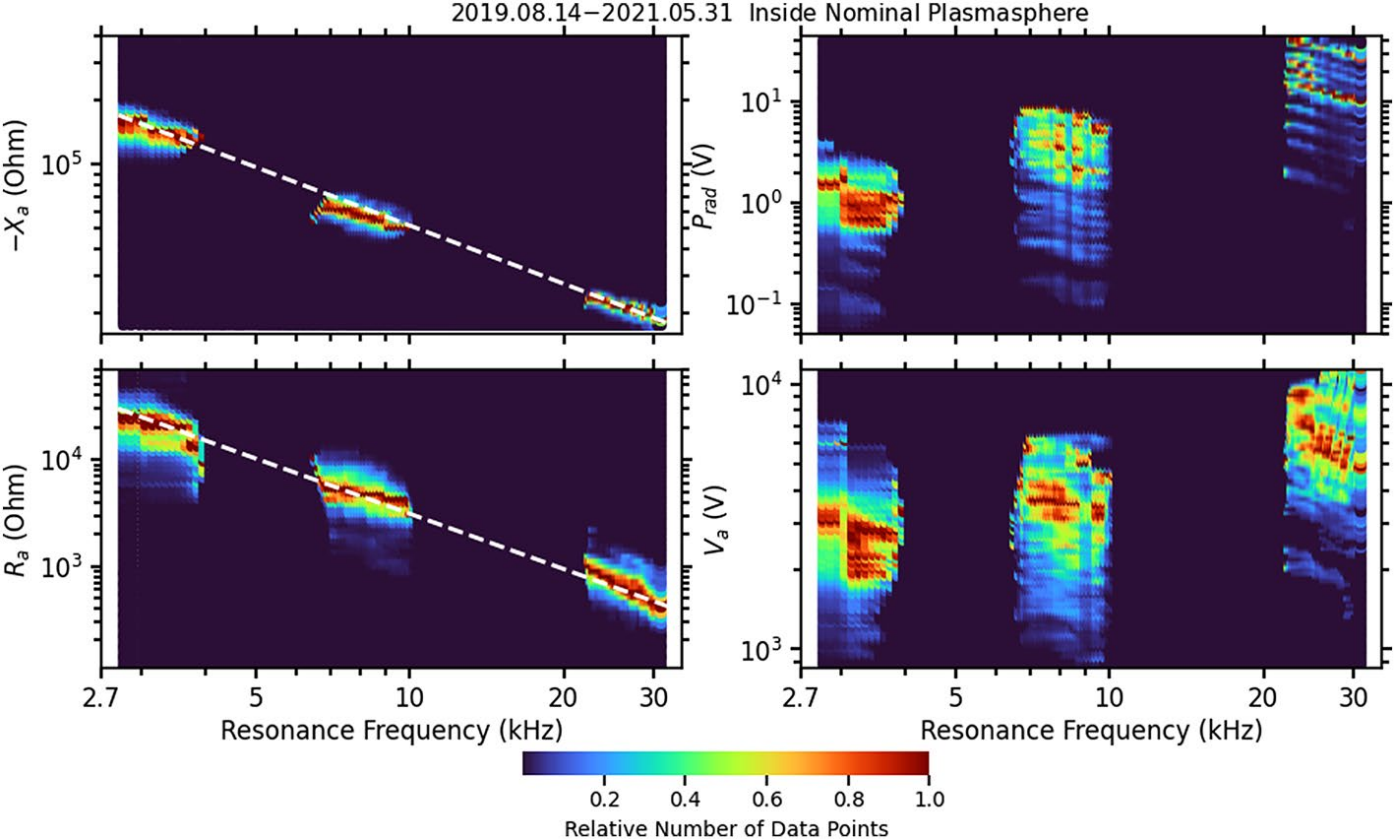
Antenna reactance –*X*_a_ (upper-left), antenna resistance *R*_a_ (lower-left), antenna power output, *P*_out_, (upper-right), and the antenna voltage, *V*_a_, (lower-right). The measurement data are grouped according to the resonance frequency into frequency bins of 100 Hz wide. The data within each frequency bin are divided according to their logarithmical value of the vertical quantity into 80 cells. The color-coding denotes the number of data points within a cell divided by the maximum number of data points in any single frequency bin. The total number of data points is about 142,700. The dashed lines in the left two panels show the best fit to a power-law correspondingly.

In the 21-month TNT transmission experiment, a total of 142,700 complete measurements made in the plasmasphere are included in this study. Figure 1 shows, from top to bottom, the derived radiation reactance, –*X*_a_, and resistance, *R*_a_ on the left panels, and power, *P*_out_, and antenna voltage, *V*_a_ on the right panels based on the method to be described in the Methodology section. *R*_a_ is the total measured antenna resistance including the radiation resistance and resistance accounting other energy losses. Since there is no evidence that the waves produced by the antenna are significantly absorbed by the plasma^22^ and the loss on the antenna is negligible, it is reasonable to treat the antenna resistance *R*_a_ as the radiation resistance *R*_rad_, and *P*_out_ as the radiated power *P*_rad_. TNT operated in three frequency bands: a low band from 2.7 to 5.7 kHz, a mid-band from 6.6 to 18.5 kHz, and a high band from 22.6 to 40 kHz; the resonances occur within each frequency band. The experiments used a large range of driving voltages of the transmitter, from 16 to 120 V although most of the transmissions were made with voltages greater than 80 V. The net antenna power output is complicated by several effects, such as tuner setting and plasma conditions. In the experiments, the internal resistance of TNT is nearly the same for each of the tuning bands but decreases from the lower band to the higher band. Therefore, the internal dissipation of TNT is greater and the antenna current is less in the lower band, see Eq. (3). This explains the step-like increase in the radiated power *P*_out_ from the lower band to the higher band in the upper-right panel of Fig. 1. Within each band, radiation resistance decreases as frequency increases, resulting in a power decrease within each band. The large spread within each frequency band in the power output is mostly due to different driving voltages.

In Fig. 1, the overall trends for—*X*_a_ and *R*_a_ are unambiguous—decreasing when frequency increases both within each band and across the three bands. Furthermore, the spread for a given frequency is not large, indicating that the impedance is not strongly dependent on driving voltage and power. A power-law fit shows that $- X_{a} \propto f_{r}^{ - 0.91}$ and $R_{a} \propto f_{r}^{ - 1.73}$, as indicated by the dashed lines. On the other hand, *P*_out_ decreases within each band but increases when going to higher bands as discussed above. Within each band $P_{out} \propto f_{r}^{ - 2.09}$. In the upper band, the radiated power can be as large as 50 W, assuming the antenna dissipation and local wave-particle resonance absorption are weak. The antenna voltage is the peak voltage from the source after the tuning and amplification measured at the antenna. It has a pattern similar to the power but mostly above kilovolts, indicating a very high voltage at the antenna relative to the satellite.

**Figure 2:**
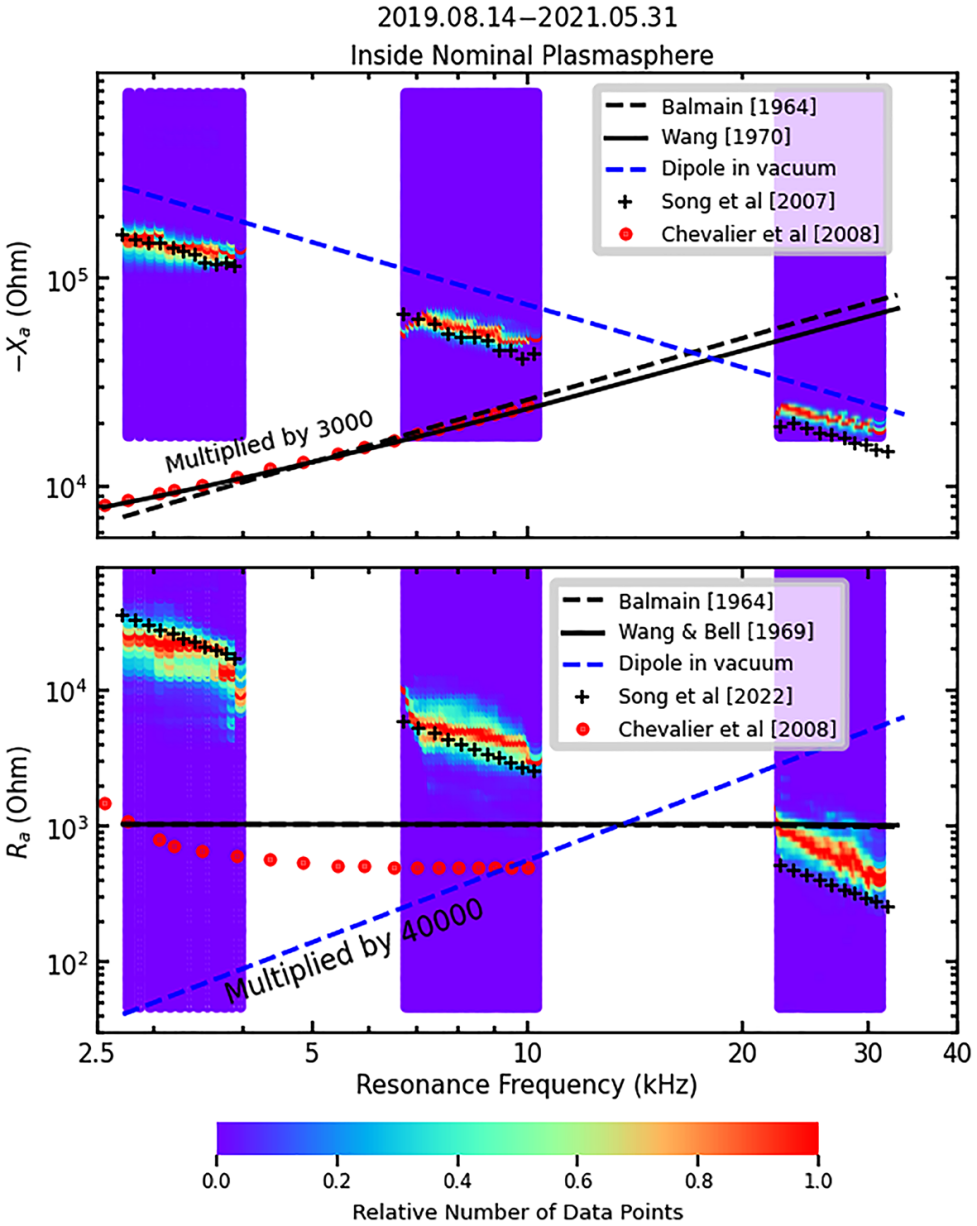
Comparison of DSX results adapted from Fig. 1 with the prediction of the radiation impedance in vacuum^18^, blue dashed-lines, Balmain model^19^, black dashed line, and Wang and Bell models^20,28^, black solid lines. Black plus-signs in the upper panel show the antenna reactance predicted by Song et al. antenna sheath model^22^ and those in the lower panel show the radiation resistance predicted by Song et al. whistler radiation model^24^.

To understand the results, in Fig. 2, we plot several theoretical model predictions on top of the observations. When calculating the theoretical values of the impedance, the physical parameters of the antenna are needed. The TNT antenna is 82 m long and consists of three separate parallel copper wires, for the purpose of redundancy and robustness, of 0.15 mm each in radius along a coilable truss system of 24 cm in diameter. In a simplified model, if each wire has a capacitance *C*’, the three parallel wires could be considered as three capacitors in parallel and the total capacitance would be 3*C*’. However, the three wires may have some interaction and the net capacitance could be represented as *αC*’ and *α* can be estimated with lab experiments. The measurements have indicated *α* = 2.2 in the vacuum of such a three-wire antenna system. Note that this factor is used for all three theoretical models and will not significantly affect the relative values among the models.

First, we compare the results to the well-understood theory of radiation in vacuum^18^ which is shown as the blue dashed lines in Fig. 2. The measured radiation resistance is more than five orders of magnitude greater than that in vacuum and shows an opposite frequency dependence. According to the theory of radiation in vacuum, wavelengths become shorter at high frequencies. As the ratio of the antenna length to the wavelength increases, so does the radiation resistance as shown in the blue dashed line in the lower-left panel. The rapid decrease of measured resistance with frequency is one of the most important discoveries from the experiment, demonstrating that the vacuum model cannot describe whistler-mode radiation in space plasma. The vacuum reactance—*X*_a_ (top panel, Fig. 2) has a similar trend to the measurements but is roughly a factor 2 larger at lower frequencies. This may be attributed to the effects of plasma interactions with the antenna.

The models of Balmain^19^, dashed black lines, and Wang and Bell^20,28^, solid black lines in Fig. 2, are quantitative models that describe the radiation reactance and resistance for whistler-mode transmission in plasma. Both are based on the “reaction method”^29^ as part of which the dot product of the radiation electric field and the current density at the antenna surface are integrated to derive radiated power and terminal impedances. Both assume time-harmonic fields and particle motions in the small-signal limit, and so do not treat wave-plasma-antenna interactions. In this low-frequency regime, they yield very similar impedance predictions. The two results agree well with each other, but they match in magnitude the resistance measurements only at the highest frequencies and do not predict any overall trend as a function of frequency. These models dramatically under-predict the observed reactance and show a frequency trend opposite to that observed. This class of models is clearly incompatible with the DSX experimental observations, and the implications of this are discussed in the next section.

To explore beyond the earlier approximations, Song et al.^22^ recently developed a physical model of the plasma sheath surrounding a VLF transmission antenna as part of the TNT development study. They reasoned that when the antenna is charged with alternately varying high voltage during transmission, as shown in the lower-right panel of Fig. 1, charged particles will move between the two antenna branches in response. When the electromagnetic field oscillates in the whistler-mode frequency range, electrons will be repelled from the negatively charged branch of the antenna and attracted to the positively charged one while the ions do not have enough time to respond before the field reverses. As a result, an ion sheath with net positive charges is formed around the negatively charged branch. Around the positive branch, on the other hand, there is a tendency to form an electron sheath with net negative charges. The overall effect of this process is that the sheath is formed around each branch of the antenna with an oscillating radius, which is achieved mostly by electron movements between the two sheaths. The corresponding electric current of such electron movements tends to cancel the driving current of the antenna, which produces difficulty for whistler-mode transmission in plasma. The Song et al. sheath model^22^ predicts that the equivalent capacitance of plasma-antenna interaction is only weakly dependent on the plasma condition and antenna current, similar to the vacuum model. The equivalent reactance of the sheath is

1 $$-{X}_{a}=\frac{1}{\alpha 2{\pi }^{2}f{\varepsilon }_{0}}\left[\mathrm{ln}\left(\frac{{I}_{a}}{{\pi }^{2}lfe{N}_{0}{r}_{a}^{2}}+2\right)-1\right]$$

where *f* is the transmission frequency, $l, \, e, \, N_{0} , \, r_{a} , \, \varepsilon_{0} ,{\text{ and }}I_{a}$ are the antenna length, elementary electric charge, plasma density, antenna radius, permittivity in vacuum, and antenna current. Factor *α* accounts for the effect of the antenna structure as described above. According to (1), the antenna reactance depends logarithmically on the plasma density. We assume that the plasma density is 2000 cm^–3^ based on measurements from the RPI instrument on the IMAGE spacecraft^30^. The prediction is shown as black plus signs in the upper panel of Fig. 2. The fluctuations in the model predictions are due to variations in the antenna current on which the reactance depends logarithmically. The model appears to be generally consistent with the measurements both in terms of magnitude and overall trend, and the performance is better than the vacuum model at lower frequencies.

Also, during the TNT development, Song et al. developed a model of whistler-mode radiation resistance^24^. It is based on the Huygens-Fresnel diffraction theory to derive the radiation from an antenna. The whistler-mode waves propagate highly anisotropic with respect to the background magnetic field. Therefore, propagation from the antenna has different speeds relative to the magnetic field. According to the Huygens-Fresnel theory, the radiation from the antenna, like from a point source, goes in any direction with the respective wavelength and propagation velocity; the wavefront of each is in the plane normal to the propagation direction. These perturbations of the radiation interfere in space at each point, e.g., an observing point. Because the propagations take different times and hence have different phases to arrive at the point, the interference can be either constructive or destructive. Strong amplitudes of waves form in limited regions in space called the Fresnel zones where perturbations interfere constructively. For the whistler mode, the Fresnel zones are two narrow regions parallel and antiparallel to the background magnetic field. The radiation cannot be observed significantly beyond the Fresnel zones because the signals either wash-out or destructively interfere. The model^24^ derives the radiation electric field and hence the Poynting vector of the radiation. The radiation resistance is derived from the summation of the radiated energy flux in the Fresnel zones with some simplifying approximations and is

2 $${R}_{rad}\approx \frac{3{\pi }^{3}{\mu }_{0}}{c}{f}_{ce}^{3}{f}_{pe}{f}^{-2}{l}^{2}=3.9\times 1{0}^{-13}{f}_{ce}^{3}{f}_{pe}{f}^{-2}{l}^{2}$$

where *μ*_0_, *c*, ${f}_{pe}$, and ${f}_{ce}$ are permeability of free space, the speed of light, electron plasma frequency, and electron gyrofrequency, respectively.

For typical conditions of DSX, *R*_rad_ is 2.6 kΩ at 10 kHz and is proportional to *f*^-−2^, similar to the observation as shown by the black plus signs in the lower panel of Fig. 2. The overall frequency dependence of power index − 2 is close to the measured − 1.73. The measured impedances, *X*_a_, and *R*_a_, are in general consistent with the theoretical predictions given in (1) and (2), supporting the validity of both the antenna sheath model^22^ and the whistler-mode radiation model^24^.

## Discussion

The first important discovery from the TNT transmission experiments is that the radiation resistance is much greater than that predicted by the widely used vacuum model. The small radiation resistance in vacuum has been the most difficult conceptual challenge for high-power VLF transmission in space because, to overcome it, a system has to employ either an extremely long antenna or an extremely large driving current. The measured large radiation resistance during the DSX experiment makes it possible to build a high-power VLF transmission system in space for radiation belt remediation purposes. As the radiation power increases with the square of the driving current, the measured radiation resistance of the order of a few kilo Ohms yields a few kiloWatts of radiated power for a 1 Ampere driving current.

The second key outcome of the DSX experiment is the unequivocal demonstration of the importance of the plasma sheath to antenna impedance when operating in the VLF regime. The confirmation of the Song et al. antenna sheath model^22^ in this study can lead to new applications since the sheath effects have generally been ignored in many plasma physics problems where quasi electric-charge-neutrality is assumed. For example, the Balmain^19^ and Wang and Bell^20^ models construct electric fields near the antenna under the assumption of time-harmonic fields and particle motions which, when inserted into the relevant equations of motion and Maxwell’s equations, do not permit particle dynamics that form sheaths. It leads to oversimplified forms of the fields near the high-voltage DSX antenna that affect the computed impedances, and in particular the reactance. Indeed, we note from Fig. 2 that the predictions from models based on this method differ dramatically from the observed reactances. It is possible that other physical assumptions in these models also contribute to the disagreement with the DSX experiment, particularly in the resistance at low frequencies, and these as well as predictions of wave normal distributions will be investigated in detail.

The final important discovery is the power-law dependence of the radiation resistance on frequency with a power index near − 2, again consistent with the Song et al.^24^ model. The general agreement between radiation resistance predictions and the experimental measurement leads to a much broader theoretical consequence because this is strong evidence confirming the approach and theoretical development based on the Huygens-Fresnel wave construction method.

So far, most of the applications of the wave construction theory are for “isotropic mediums”, such as air or vacuum. For example, the Fresnel zone concept is currently used in cellphone relay tower designs, in radar designs, and in optics. The Song et al.^24^ model shows that the theory can be used for an anisotropic medium within which multiple anisotropic wave modes can propagate. The successful confirmation of the approach and technical development opens the possibilities for any radiation problem in anisotropic mediums. There are many such mediums and there are multiple wave modes that can propagate in such a medium, e.g., space plasma and astronomical/astrophysical systems. For example, the radio emission from a pulsar may be considered radiation in a magnetized plasma. There are also magnetized plasma experiments in laboratories. For example, in controlled fusion experiments, injecting wave energy into plasma is an active research subject, and whistler-mode waves are among the important possibilities to pump energy into plasma. Even in optics, there are anisotropic mediums such as birefringent mediums, to which Fresnel zone construction theories can be developed if the light source is embedded in the medium.

On the application side, there are several groups in the world that are considering developing high-power whistler transmission systems in space for various purposes. In addition to radiation belt depletion, for example, one may envision the geomagnetic field lines to be used for long-range inter-hemispherical communication based on the Fresnel zone concept.

We note here that although the Song et al. whistler-mode radiation model^24^ is generally consistent with the experimental results, more improvement may be needed as it includes some simplifying approximations. An active research subject is how to reduce the approximations.

There is a question of whether the measured transmission power is the radiation power given by the theory of radiation resistance. For example, it is possible that there is strong dissipation in the plasma near the antenna. If this is true, the direct comparison of radiation resistance made in Fig. 2 between the transmitter output and theoretical radiation is not valid. However, if the dissipation around the antenna is strong, the plasma is under forced oscillations. The electromagnetic field associated with such oscillations can be easily detected and identified by the TNT sounding measurements. No evidence for such oscillations and hence locally enhanced dissipation has been seen. Therefore, the local dissipation is unlikely to be substantial.

It is conceivable that TNT does not transmit wave power far from the antenna, so this possibility was tested via an experiment conducted between two satellites^31^. In this experiment, TNT transmitted a distinct pattern of signals to the JAXA Arase satellite^32^, which received the transmission unambiguously about 1100 km away from DSX when the two satellites were nearly along the same geomagnetic field line. In addition, the DSX broadband receiver (BBR) instrument often received delayed echoes of the radiated waves when the DSX TNT was in operation. The BBR measured echoes will be reported separately. Hence far-field propagation of the TNT transmissions has been confirmed.

## Methods

**Figure 3:**
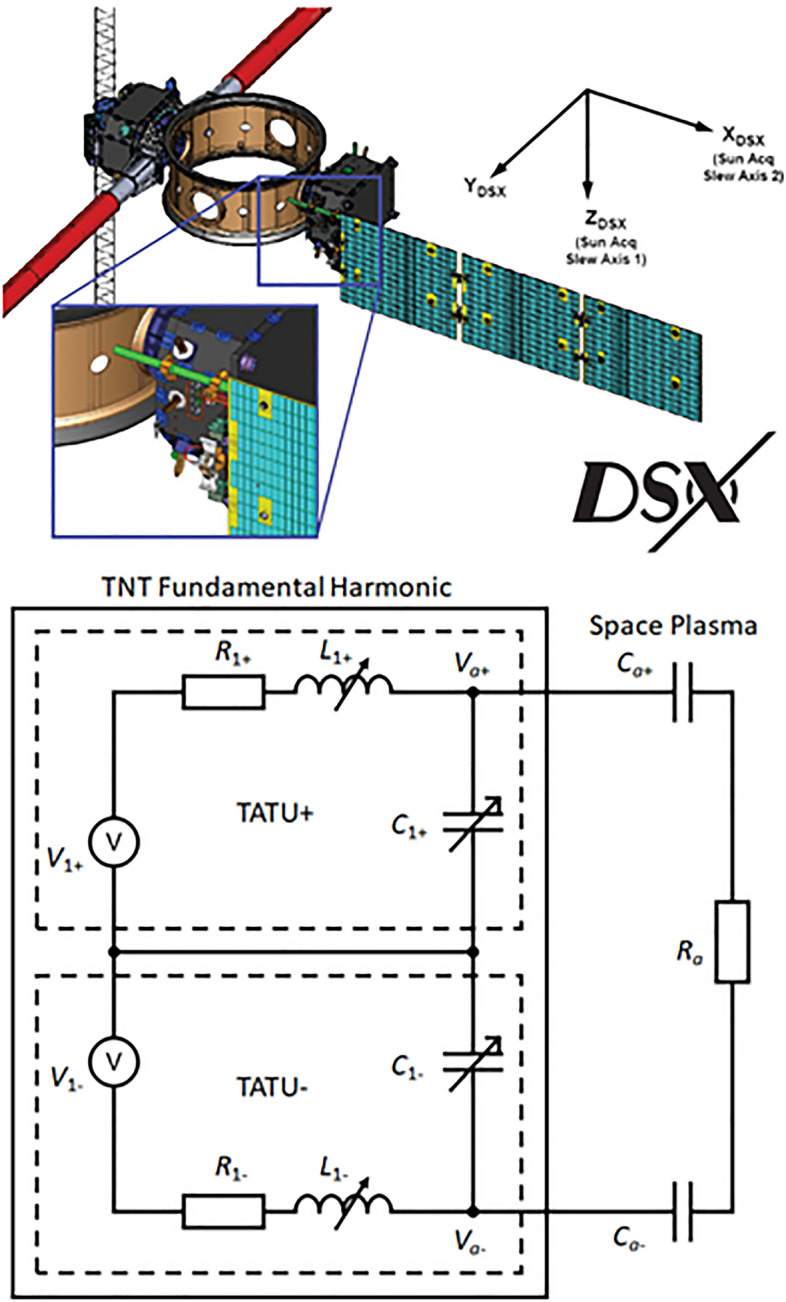
(**a**) The structure of DSX satellite, https://directory.eoportal.org/web/eoportal/satellite-missions/content/-/article/dsx. The two branches of the TNT antenna are shown as the red cylinders, 40 m long each side separated by 2 m diameter satellite body. (**b**) Equivalent circuit of TNT presented in this report.

**Figure 4:**
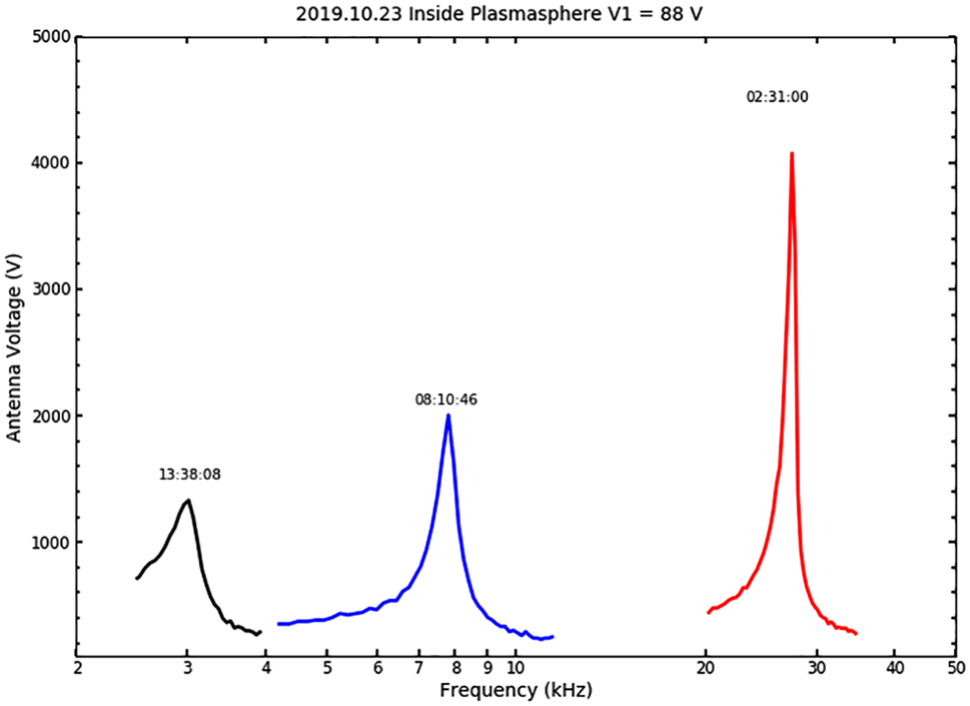
An example of resonance curves, antenna voltage of Y+ branch, *V*_1+_ as functions of frequency, from high-power transmission (*V*_1±_  = 88 V) in a nominal plasmasphere determined by the Carpenter-Anderson empirical plasmaspheric model^34^. The transmission started in the higher frequency band and then went to mid and lower bands. The universal time when these voltage curves were acquired is indicated above each curve.

Figure 3 shows (a) the DSX structure and (b) the TNT equivalent circuit. In TNT, the Digital Control Unit (DCU) generates the time sequence of the desired frequency and waveform, as indicated by V in Fig. 3b. This signal is sent to two Tuner-Amplifier-Transmission Units (TATU), denoted by subscripts + and –, respectively, and is amplified to *V*_1±_. Subscript 1 denotes the fundamental frequency. Each TATU, in addition to the power amplifier, consists of an adaptive tuner with a bank of capacitors, *C*_1_, and inductors, *L*_1_ which has an internal resistance, *R*_1_, providing 4096 combinations of L-C values. The signal, after transiting the tuner, is then fed into the antenna. The voltage at each antenna terminal relative to the ground of TATU is measured. In this study, the two TATUs are set with 180° phase difference, i.e., $V_{1} = \left| {V_{1 + } } \right| + \left| {V_{1 - } } \right|$. TNT is also able to transmit the third harmonics with the correct phase and amplitude so that the waveform can mimic a square wave. In this study, since the radiation impedance is a function of space plasma conditions, TNT is set at a given *L*_1_, *R*_1_, and *C*_1_ and transmits first in a large frequency range with a coarse frequency step, aiming at finding the frequency in which the TATU and plasma conditions are tuned, and then successively reduces the frequency range for fine-tuning. Figure 4 shows an example of measured resonance curves which is the antenna voltage as a function of frequency for each of the three operation frequency bands on the same day inside the radiation belts.

From each resonance curve, three quantities are derived: the tuning resonance frequency, *f*_r_, where the voltage reaches the maximum, peak voltage value, *V*_a_, and half-power frequency width, Δ*f*, which is the frequency span between two points where the voltage drops to $1/\surd 2$ *V*_a_ (i.e., at half-power points). The resonance occurs when the frequency satisfies $\left( {2\pi f_{r} } \right)^{2} = 1/LC$ where *L* and *C* are the total equivalent inductance and capacitance of an R-L-C circuit. The quality factor $Q = f_{r} /\Delta f = 2\pi f_{r} L/R$ of the circuit is derived, where *R* is the total resistance. From Fig. 3b, the antenna reactance *X*_a_, capacitance, *C*_a_, resistance, *R*_a_, and power delivered to the antenna, *P*_out_, can be determined with the system and measured parameters

$$-{X}_{a}=\frac{1}{2\pi {f}_{r}{C}_{a}}=\frac{2\pi {f}_{r}{L}_{1}}{1-{\left(2\pi {f}_{r}\right)}^{2}{L}_{1}{C}_{1}}$$

3 $${R}_{a}=\left(\frac{2\pi {f}_{r}{L}_{1}}{Q}-{R}_{1}\right){\left(1+\frac{{C}_{1}}{{C}_{a}}\right)}^{2}$$

$${P}_{out}=\frac{1}{2}\frac{{V}_{a}^{2}{R}_{a}}{{R}_{a}^{2}+{X}_{a}^{2}}=\frac{1}{2}{I}_{a}^{2}{R}_{a}$$

where $L_{1} = L_{1 + } + L_{1 - }$, $C_{1} = C_{1 + } C_{1 - } /(C_{1 + } + C_{1 - } )$, $R_{1} = R_{1 + } + R_{1 - }$, $C_{a} = C_{a + } C_{a - } /(C_{a + } + C_{a - } )$, $V_{a} = V_{a + } + V_{a - }$, and ${I}_{a}={V}_{a}/\left({R}_{a}^{2}+{X}_{a}^{2}\right)$ is the system current flowing to the antenna. The driving voltage of both TATU branches *V*_1±_ is the same but opposite in phase for this experiment. In the case shown in Fig. 4, the driving voltage is at 88 V or 176 V peak-to-peak amplitude. The antenna peak voltage increases to approximately *Q* times *V*_1±_. For example, the antenna voltage amplitude in the high-band is about 4500 V or 9000 V peak-to-peak amplitude. The lower band has a broader resonance curve and hence a smaller *Q*.

During the DSX mission, a total of 488,830 resonance curves were collected from each antenna branch. About 39% of them are when one of the antenna branches was turned off during the experiment or the resonance curve has more complicated features than a single peak between half-power points or one of the half-power points is out of the frequency range of the specific band, so that the *Q*-factor cannot be meaningfully determined. About another 6% of the total involved transmission of the third harmonics which may interfere with the determination of the fundamental frequency in this study. There are also some anomalous cases, about 9%, when the resonance frequency and bandwidth from two antenna branches are very different and different from neighboring measurements. After excluding those three types of events, resonance curves are available from each of the two branches for this study.

To double-confirm the power measurements shown in Fig. 1, we show the direct measurements of the antenna voltage in the lower-right panel. Given that − *X*_a_ is much greater than *R*_a_ and that − *X*_a_ ~ 1/*f* and *R*_a_ ~ 1/*f*^2^, from the last expression in (3), *P*_out_ should be approximately proportional to *V*_a_^2^. This is confirmed by the similarity of the right two panels of Fig. 1.

Because the most dangerous high energy electrons (> 500 keV) by virtue of a long lifetime (> 100 days) are trapped in the region below L-shell of L = 3^34^ which is inside so called the plasmasphere^2^, for the purpose of remediation of radiation belts, we are most interested in transmissions in the plasmasphere. Nevertheless, we did analyze the data outside the plasmasphere. The results are similar to those shown in Figs. 1 and 2 with the fewer data points due to the DSX orbit and DSX TNT operational schedules. We use the Carpenter–Anderson plasmaspheric model^33^ to select the transmission events in the plasmasphere. There are about 142,700 resonance curves satisfying these conditions, shown in Fig. 1. The data are binned with 100 Hz in frequency and 80 bins for each of the three parameters,—*X*_a_, *R*_a_, and *P*_out_. The color coding presents the relative number of events in each frequency and parameter bin.

## Summary

The DSX-TNT instrument with an 82-m tip-to-tip antenna has been used to successfully conduct a series of high-power VLF transmission experiments. The radiated power can be as high as 50 W. This is unprecedented. To put this in perspective, the Radio Plasma Imager (RPI) transmitter^35,36^ with a 500 m long tip-to-tip antenna on the NASA IMAGE satellite also transmitted in the VLF frequency range. When scaled to the DSX antenna length, the radiated power would have been 0.25 W. The large radiation resistance, of the order of kiloOhms, removes the assumption that the antenna must be extremely long, or that the driving current need to be prohibitively large, in order to build a high-power VLF transmission system in space.

The experimental result demonstrates the key role played by the plasma-antenna-wave interaction in the radiation of whistler-mode waves in space plasma, showing that radiation theories that omit it cannot accurately predict the antenna impedance. It lends strong support to the expressions of (1) and (2) proposed by Song et al.^22,24^. An immediate consequence is that, according to Eq. (2), the radiation resistance increases with the antenna length and electron gyro and plasma frequencies; this raises a favorable possibility to place the transmitter in low-Earth orbit (LEO). The gain in the resistance can be used to reduce the length of the antenna. Therefore, a shorter antenna in LEO may be a realistic option for radiation belts remediation.

The confirmation of the Song et al.^24^ radiation model has a much broader consequence, in addition to the future development of space-based VLF capabilities, because this theory can be quantitatively applied to other radiation problems in anisotropic mediums or anisotropic propagating modes, which may be found in space, astrophysical systems, laboratory plasma experiments, and optics.

## Data Availability

The data produced in this study is available online https://ulcar.uml.edu/downloads.html/DSXscientificreports.
